# Lessons From the Community-Centered Health Home Demonstration Project: Patient-Centered Medical Homes Can Improve Health Conditions in Their Surrounding Communities

**DOI:** 10.5888/pcd13.160262

**Published:** 2016-08-04

**Authors:** Daphne Miller, Eric T. Baumgartner

**Affiliations:** Author Affiliations: Eric T. Baumgartner, Louisiana Public Health Institute, New Orleans, Louisiana.

The latest challenge for health care is to improve population health and well-being while continuing to care for individuals. In the United States, good health depends more on living conditions and work environment than on access to medical services (1). However, only recently has the health care system made population health and health promotion a priority (1). This historic shift in the system is in response to incentives within the Affordable Care Act to meet its triple aim: improving quality of care and patient satisfaction, improving the health of populations, and reducing the per capita cost of health care.

 Local and state health partnership models, such as accountable care organizations and accountable communities for health, are being tested, and large health care organizations are embracing a “health in all policies” approach, which emphasizes multisector collaboration to improve population health (2,3). These innovations are encouraging, but there are no concrete strategies for how primary care clinics, and safety-net clinics in particular, can participate in this larger effort (4). We propose that the medical home model — a system of primary care designed to meet the needs of individual patients by delivering coordinated and accessible services — offers a ready framework for community clinics to offer primary prevention to individuals and to improve health at the population level.

Five safety-net clinics in 4 US Gulf Coast states (Louisiana, Mississippi, Alabama, and Florida) are participating in the Community Centered Health Home Demonstration Project, directed by the Louisiana Public Health Institute, to expand from a patient-centered medical home (PCMH) to what is being called a community-centered health home (CCHH) (5). The CCHH model provides a framework for primary care – and health care organizations in general – to address individual health needs while systematically addressing community conditions that affect individual health. This article describes the experience of clinics in the Gulf Coast states in creating a CCHH by building on the 3 established characteristics of a successful PCMH: 1) an explicit vision for how to serve a population, 2) engaged and visible leaders, and 3) effective clinical teams (6).

## Explicit Vision for How to Serve the Population

All demonstration clinics had an established social mission, and yet the broadening of their focus from individual patient needs to the community’s needs changed their approach to illness and injury prevention. As documented in previous reports, the PCMH structure increased patient access to secondary prevention and screening services, such as mammograms and blood pressure screening, and to lifestyle and chronic disease self-management education (7,8). The CCHH approach takes this structure a step further by acknowledging that socioeconomic and environmental factors greatly influence behavior and disease risk and that these broader influences must therefore be targeted in tandem with efforts directed toward the individual’s health (9). To expand from health education and screening to preventing chronic disease from occurring in the first place, CCHH sites are forging partnerships with public health and other organizations dedicated to improving community conditions that broadly influence health. In addition, they are co-locating with social services such as the Special Supplemental Nutrition Program for Women, Infants, and Children (WIC), insurance enrollment, and legal assistance so that community members can access a variety of services at one site. For example, the Escambia Clinic, a CCHH demonstration site in Pensacola, Florida, relocated from a stand-alone clinic building to an elementary school and is using its influence and visibility to affect local issues such as ensuring safe housing for low-income residents, nutritious food in schools, and environmental justice. Although most clinicians and administrators at Escambia and other CCHH sites long understood how social determinants affected the health of their patients, the expanded vision for the clinic allows them to become active participants in improving these conditions.

## Engaged and Visible Leaders

Medical directors and executive directors at each of the 5 demonstration sites play a key role in helping their clinics expand their focus to include people who access their services *and* the larger community infrastructure. In a May 2016 conversation, Chandra S. Smiley, executive director of the Escambia clinic stated, “We don’t just want to be the community clinic. We want to be the community*’s* clinic.” Smiley explained that although her clinic historically operated within its walls and delivered services to individual patients, she now expects it to leverage its reputation and its human and financial resources to influence health conditions in the entire community.

CCHH clinic leaders introduce their agenda into clinic programs and policies and encourage all staff members to share their approach. They also use their visibility in the community to advance the idea that a clinic can initiate partnerships with stakeholders beyond those involved in health care to promote health. To prepare leaders for this role, the Gulf Coast states project developed a curriculum and facilitated peer-to-peer mentorship between CCHH clinic directors, 2 knowledge-sharing practices that are also used to facilitate adoption of the PCMH model.

## Clinical Teams

CCHH clinics take advantage of the collaborative team structure that is the central part of the PCMH model. All team members, from physicians and nurses to clerical staff, understand how environmental and social factors shape people’s health, and each has a role in addressing those factors. Depending on skill sets, team member involvement in the CCHH structure falls into one or more of the following categories: 1) advocacy, 2) information sharing, or 3) coordination with community partners. One common scenario is for clinicians to identify the most prevalent health issues they treat in the clinic and trace them back to their community source. (The recent initiative to introduce a population health curriculum into medical schools, a curriculum which emphasizes integrating public health and clinical care skills, will help prepare future physicians for this type of work.) (10) Examples are asthma triggered by substandard housing conditions or toxic environmental exposures and diabetes and obesity linked to the scarcity of healthful food and to unsafe streets and parks. Next, clinicians share this information with other agencies and advocate for policies and programs to change the physical and social environment. Clinic staff members at some demonstration sites became involved with efforts to limit the density of fast food outlets and liquor stores, to construct safe and affordable housing, and to increase access to green space; some also joined initiatives to raise the minimum wage. Similarly, staff members with information technology expertise pooled clinical data, blending it with data from outside agencies and performing geospatial analyses, an approach that is increasingly used to help identify the root causes of disease patterns in a community (11). Health coaches and community health workers who were part of the existing PCMH team continue to serve individual patients, but now their role has expanded. They also act as liaisons between the clinic, the public health department, and other community stakeholders to prevent program redundancy and to create multisector health partnerships.

A wide-scale shift is under way in health care in the United States, from focusing solely on the individual to also focusing on population health; from delivering health care to also working on advocacy and community engagement. Recent experience with 5 CCHH demonstration clinics in the Gulf Coast states indicates that clinics can make these changes by building on the core characteristics of the PCMH and by becoming CCHHs. Additional research is needed to find ways that fully support this new approach. Research should not focus only on outcome indicators, such as quality of care and cost, but also on process indicators such as the number and quality of partnerships in which the clinic is a stakeholder. In addition, new national, state, and local policies are needed to finance this population health approach. For example, the billions of community benefit dollars from nonprofit hospitals, which are now overwhelmingly used to cover uncompensated medical care, could be earmarked specifically for improving community infrastructure (12). Although the CCHH model is still in a demonstration phase, the growing emphasis on pay for value and on expanding health care’s role in population health will generate interest in this approach across the country.
