# Erratum, Vol. 13, August 4 Release

**DOI:** 10.5888/pcd13.160262e

**Published:** 2016-09-15

The authors inadvertently omitted a funding source from the Acknowledgments section of the article “Lessons From the Community-Centered Health Home Demonstration Project: Patient-Centered Medical Homes Can Improve Health Conditions in Their Surrounding Communities,” which was released on August 4, 2016. The following information was added on August 30, 2016:

The Louisiana Public Health Institute’s Community-Centered Health Home Demonstration Project is supported through the Gulf Region Health Outreach Program (GRHOP). The GRHOP is funded from the Deepwater Horizon Medical Benefits Class Action Settlement, which was approved by the US District Court in New Orleans on January 11, 2013.

The corrected article appears online at http://www.cdc.gov/pcd/issues/2016/16_0262.htm. We regret any inconvenience this omission may have caused.

